# From Waste to Catalyst: Cobalt-Functionalized Silica as a Photocatalyst for Water Purification

**DOI:** 10.1021/acsomega.6c00378

**Published:** 2026-07-17

**Authors:** Saša Zeljković, Andraž Šuligoj, Milica Kosić, Goran Dražić, Alenka Ristić, Nataša Zabukovec Logar, Nataša Novak Tušar

**Affiliations:** † University of Banja Luka, Faculty of Natural Sciences and Mathematics, Department of Chemistry, Mladena Stojanovica 2, Banja Luka 78 000, Bosnia and Herzegovina; ‡ National Institute of Chemistry, Department of Inorganic Chemistry and Technology, Hajdrihova 19, Ljubljana SI-1001, Slovenia; § University of Ljubljana, Faculty of Chemistry and Chemical Technology, Večna Pot 113, Ljubljana SI-1000, Slovenia; ∥ National Institute of Chemistry, Department of Materials Chemistry, Hajdrihova 19, Ljubljana SI-1001, Slovenia; ⊥ University of Nova Gorica, Graduate School, Vipavska 13, Nova Gorica SI-5000, Slovenia

## Abstract

The repurposing of waste industrial materials for use in environmental remediation is of great interest in terms of the circular economy as well as public health and safety efforts. Herein, an innovative approach is presented to synthesize a cobalt-functionalized waste silica (Co-MS) utilizing a green, solvent-deficient method (SDM), using industrial waste micron-sized silica (MS) as a sustainable support material. The resulting Co-MS composite is evaluated as a photocatalyst for the removal of methylene blue (MB) under natural sunlight, offering an energy-efficient and environmentally friendly treatment method. Comprehensive characterization confirmed the formation of multiple-valence-state cobalt nanoparticles—Co, CoO, and Co_3_O_4_ on the waste silica support. The resulting optimal composite includes 9.1 wt % cobalt oxide species and shows a polyoxide nature with finely dispersed cobalt oxide nanoparticles that are less aggregated compared to bulk Co_3_O_4_ and show an improved photocatalytic performance under solar light. Incorporation of cobalt oxide species into MS also results in an *S*_BET_ increase from 43 m^2^ g^–1^ for bulk Co_3_O_4_ to 91 m^2^ g^–1^ for the Co-MS composite. The selected composite Co-MS thus achieves 87.5% MB removal efficiency within 1 h, demonstrating its potential as a low-cost, sustainable photocatalyst for wastewater treatment applications.

## Graphical abstract

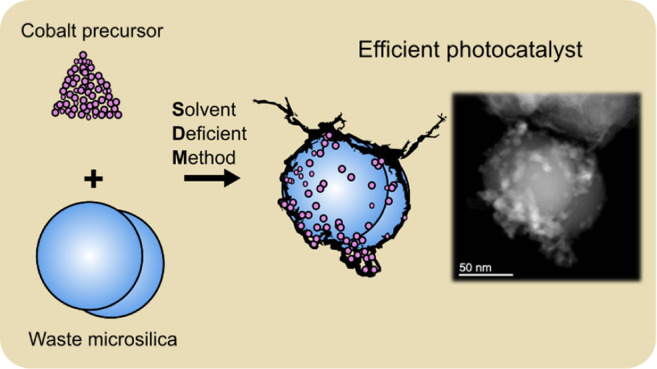

## Introduction

Clean drinking water is becoming increasingly polluted due to industrial growth and rising demand for various materials and goods.^1^ Developing accessible synthesis methods for visible-light-activated nanomaterials is crucial, as these materials are promising candidates for photocatalytic applications because of their efficient photon absorption and subsequent use in redox reactions.^2^ Organic dyes, as significant pollutants with low rates of natural degradation, can be efficiently broken down into CO_2_ and H_2_O through photocatalysis.^3^

Methylene blue (MB) is an aromatic heterocyclic basic dye^4^ and is one of the most widely used dyes utilized in textile dyeing, biological staining in laboratories, and as a photosensitizer in photodynamic cancer therapy.^5^ When released into the environment, it can harm aquatic life and human health, causing acute exposure symptoms such as nausea, vomiting, sweating, and disorientation.^6^ In wastewater, MB poses significant environmental risks. It can inhibit the growth of algae essential for oxygen production in water^7^ and harm microbes and aquatic organisms while also exhibiting a low rate of natural degradation.^8^ Among all current technologies for degrading industrial dyes, photocatalysis is particularly effective for degrading MB and other pollutants.^9^

The circular economy model is a sustainable production strategy that emphasizes the reuse of waste as secondary raw materials, enabling comprehensive waste management. At the European level, this model is implemented through the Action Plan for the Implementation of the Circular Economy by the European Parliamentary Commission,^10^ which presents significant opportunities in material design, including the design of photocatalysts. While photocatalytic materials are promising for degrading MB on their own, immobilizing them on a suitable support often provides benefits such as increased surface area, reduced leaching, easier postreaction separation, etc.^11^ Metal production industries generate substantial amounts of byproducts from silicon production, such as waste micron-sized silica (MS). MS is a fine, semicrystalline SiO_2_ produced during silicon manufacturing,^12^ in which quartz is heated to approximately 2000 °C with the aid of a carbon source that reduces silica to silicon. Small amounts of silicon vapor are released from the furnace, oxidized, and condensed to form microspheres, which are collected using fans and bag filters. MS is widely recognized for its beneficial applications in concrete^13^ and is being explored as a base for lubricants.^14^

Cobalt-based nanostructures possess attractive physicochemical properties suitable for photocatalytic applications. Notably, they feature a tunable direct optical band gap that allows utilization of either the UV or visible region of the solar spectrum.^15,16^ In recent years, researchers have explored various methods for synthesizing Co-based nanoparticles, emphasizing scalability and eco-friendliness.^17^ Despite its many advantages, Co_3_O_4_ has limitations, including low solar energy response and a high electron–hole recombination rate.^18^ To address these challenges, supported catalyst systems and heterojunction construction are widely used strategies, as they increase the specific surface area—enhancing the availability of active sites—and improve electron–hole separation,^19^ thereby boosting photocatalytic efficiency.^18^ The Co_3_O_4_–SiO_2_ system has been studied for both catalytic^20,21^ and photocatalytic applications.^22^ Additionally, mixed CoO-Co nanoparticles dispersed on a SiO_2_ support have demonstrated high activity in the Fischer–Tropsch reaction, the water–gas shift reaction, and hydrogenation reactions.^23^

In this study, we focused on synthesizing Co-MS composite using a green solvent-deficient method (SDM)^24,25^ as effective photocatalysts for MB degradation, offering a practical and sustainable approach to wastewater treatment.

## Experimental Section

The materials used for the synthesis of bulk Co_3_O_4_ and Co-MS composite were ammonium hydrogen carbonate (NH_4_HCO_3_ 99%, Lach Ner) and cobalt nitrate hexahydrate (Co­(NO_3_)_2_·6H_2_O 99%, VWR Chemicals BDH), and MS (Metalleghe Silicon company, Bosnia and Herzegovina). Ammonium hydrogen carbonate and cobalt nitrate hexahydrate were mechanically stirred (using a mortar and pestle) for 15 min at a 2:1 molar ratio, following previous methods.^24–26^ In the case of the composites, the required amounts of MS were added at this stage. Several weight ratios of Co_3_O_4_ in the microsilica composites were synthesized: 0.025 (S1), 0.05 (S2), 0.1 (S3), 0.15 (S4), and 0.20 g (S5) of Co_3_O_4_ per 1 g of MS. The products were further washed with 150 mL of distilled water to eliminate NH_4_NO_3_ byproduct from the reaction mixture. The resulting sample was then filtered through a Büchner funnel with blue stripe filter paper and left to dry in an oven for 20 h at 50 °C. After drying, the sample was homogenized to achieve further particle reduction in a mortar and subsequently calcined at 300 °C for 2 h at a 10 °C·min^–1^ heating/cooling rate in a muffle furnace (Nabertherm, Lilienthal, Germany). A photocatalytic screening test (Figure 11b) showed that the S3 composite exhibited the best weight-to-performance ratio; hence, the majority of characterization techniques described below were applied to this composite.

For thermal analysis, the intermediate product samples (collected before calcination) were used. Specifically, the Co_3_O_4_ intermediate product (12.831 g) and the S3 intermediate product (24.944 g) were heated under a nitrogen atmosphere at a rate of 10 °C·min^–1^ up to 500 °C using a TGA DSC Setaram Setline thermal analyzer. Before analysis, the MS sample was also dried in an oven at 50 °C for 20 h and homogenized using a mortar.

X-ray powder diffraction (XRD) patterns were recorded on an X’Pert PRO (PANalytical, The Netherlands) high-resolution diffractometer using Cu*K*α_1_ radiation (λ = 1.5406 Å).

Field-emission scanning electron microscopy (FE-SEM) images were recorded by using a ThermoScientific Apreo 2 instrument with an acceleration voltage of 2.0 kV and a working distance (WD) of 9.1 mm. Transmission electron microscopy (TEM) investigations were performed using an atomic resolution scanning transmission electron microscope (AR STEM, JEOL ARM 200 CF) equipped with high-angle annular dark field scanning transmission electron microscopy (HAADF-STEM) imaging. Selected-area electron diffraction (SAED) patterns were collected to analyze the crystal structure and orientation relationships. Elemental analysis and mapping were carried out by combining energy-dispersive X-ray spectroscopy (EDXS) with electron energy-loss spectroscopy (EELS).

The N_2_ adsorption–desorption measurements were performed using a 3P Instruments surface area and pore size analyzer, Sync 220A model. Before analysis, samples were degassed at 120 °C for 4 h under vacuum.

The attenuated total reflectance FT-IR/ATR spectra were recorded on a PerkinElmer spectrometer (Spectrum 100, USA) using a MIR TGS detector. Spectra were recorded from 4000 to 400 cm^–1^ with a resolution of 2 cm^–1^ using a diamond crystal in a horizontal position. The Raman analyses were performed using a Bruker Senterra dispersive Raman microscope with a laser excitation wavelength of 785 nm (Nd:YAG) at a total emission power of 1 mW and an accumulation time of 50 s. The samples were examined as raw powder materials under a BX51 optical microscope coupled with a Raman spectrometer and examined by focusing the laser beam with a 100× objective lens. The spectra were recorded using a CCD detector and a 400 lines·mm^–1^ or 1200 lines·mm^–1^ diffraction grating (spectral resolution 4 cm^–1^ or 1.5 cm^–1^).

Diffuse reflectance spectra (DRS) of the solid catalysts were recorded on a Lambda 650 (PerkinElmer) spectrometer in reflectance mode (*R*_∞_) in the range of 200–900 nm, using Spectralon as a 100% reflectance standard. The data were transformed using the Kubelka–Munk theory (eq 1) and subsequently plotted using a Tauc plot (eq 2).^27^

1 $$F\left(R_{\infty }\right)=\frac{{\left(1-R_{\infty }\right)}^{2}}{2R_{\infty }}$$

2 $${[F\left(R_{\infty }\right)hv]}^{1/n}=A\left(hv-E_{g}\right)$$

where *h* is Planck’s constant, *A* is the absorption constant, ν is the light frequency, *E*_g_ is the band gap energy, and *n* is related to the type of electronic transitions: 1/2 for direct and 2 for indirect band gap. Deconvolution and fitting of the DRS spectra, transformed according to the Kubelka–Munk theory, were carried out using the Fityk 1.3.1 software with a Gaussian function.

For photocatalytic experiments, 100 mL of MB solution (10 mg·L^–1^) was combined with the designated mass of photocatalyst in separate beakers. The photocatalyst mass was optimized to increase the efficiency of the photocatalytic degradation of MB by using photocatalyst quantities of 50, 100, and 150 mg. All systems were kept in the dark for 1 h to achieve adsorption–desorption equilibrium before being exposed to natural sunlight (location: 44°46′44.6″ N, 17°11′56.4″ E) in July 2024 under clear and sunny sky conditions, and under constant stirring using a magnetic stirrer. During the experiments, the solar light intensity ranged from approximately 720 to 880 W·m^–2^, estimated from measured illuminance of 90000–110000 lx (Lutron LX-101 LIGHT METER) using a daylight conversion factor (1 W·m^–2^ ≈ 125 lx)^28^ Specifically, the light intensity was continuously monitored and recorded at 30 min intervals throughout the experiment. A limitation of this study is that the results cannot be directly compared with those obtained using standard solar simulators. Samples (2.5 mL) were collected in triplicate at 10 min intervals over 180 min. The temperature was maintained at 25 ± 2 °C throughout the experiment using a constant-temperature water bath. For spectrophotometric determination of the concentration of MB solutions after decantation, absorbance at 663 nm was measured using a UV–vis LAMBDA 25 PerkinElmer spectrophotometer. A calibration curve was generated from the absorbance values of standard MB solutions with concentrations of 1, 3, 5, 7, 9, and 12 mg·L^–1^. A dark adsorption experiment lasting 180 min was conducted using the same sample conditions as the photocatalytic tests. The MB adsorption results for MS and the S3 composite were evaluated under these conditions.

## Results and Discussion

Bulk Co_3_O_4_ and Co-MS composites are synthesized by using a green SDM, providing a practical and sustainable method for nanomaterial fabrication. The SDM approach is notable for its environmental benefits, simplicity, and efficiency in producing nanostructured oxides.^26^ Many of these oxides are considered promising photocatalysts for wastewater treatment, particularly for the degradation of MB.^29^ Unlike conventional physical and chemical synthesis methods, which often require high energy input, long processing times, and hazardous solvents, SDM offers a more eco-friendly and scalable solution. The combination of nano- and microscale oxide materials within the composite, along with macro- and mesoporous structures, underscores the significance of the synthesis strategy described here, as these hierarchical features are essential for advanced catalytic and related applications. Photocatalytic activity screening (see below) showed that the Co-MS composite with 9.1 wt % cobalt oxide species had the highest performance-to-price ratio; therefore, the characterization presented below is based on this sample.

### Characterization

The TG/DSC curves for the Co_3_O_4_ precursor mixture, the S3 Co-MS composite mixture, and MS obtained by heating to 500 °C at a rate of 10 °C·min^–1^ in a nitrogen atmosphere are presented in Figure 1.

**Figure 1:**
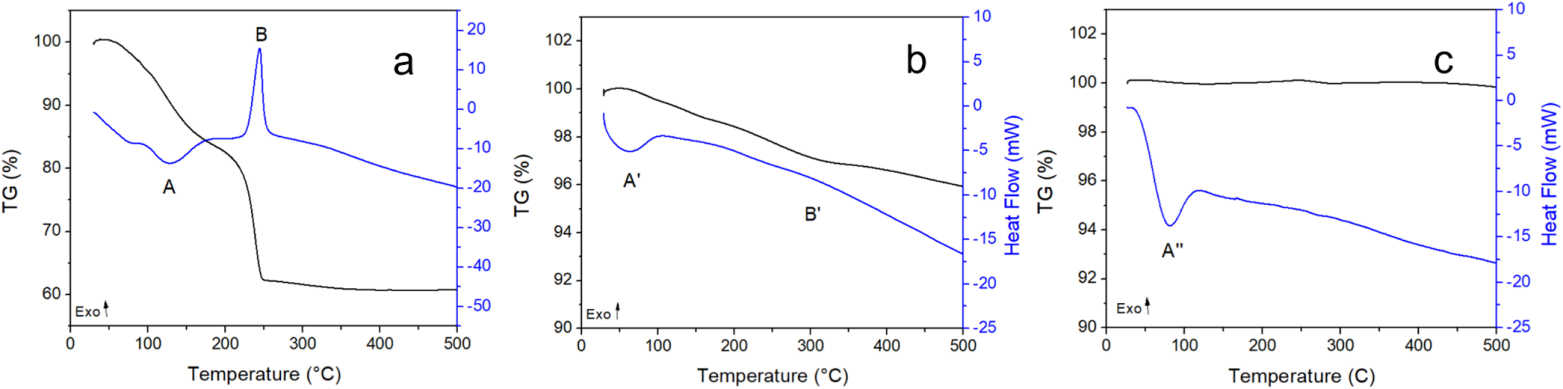
TG/DSC measurements of a) the Co_3_O_4_ precursor mixture, b) the Co-MS composite precursor mixture, and c) MS obtained by heating to 500 °C at a rate of 10 °C·min^–1^ in a nitrogen atmosphere. The black line represents the thermogravimetric curve (TG), whereas the blue line (DSC) monitors endothermic and exothermic changes caused by heating.

During precursor mixing for 15 min at room temperature, a mechanically induced reaction occurred between cobalt nitrate hexahydrate and ammonium bicarbonate. In a previous study performed by Smith et al.,^24^ the material obtained after mechanical mixing and drying consisted of Co_2_(OH)_3_(NO_3_)_3_, Co_2_(OH)­(NO_3_)·H_2_O, Co­(OH)_2_, and NH_4_NO_3_. Washing with 150 mL of distilled water prevented the formation of certain intermediate reaction products. As previously determined,^30^ rinsing before annealing prevents Co_2_(OH)_3_(NO_3_)_3_ and Co_2_(OH)­(NO_3_)·H_2_O from forming (the nitrate ligand substitutes for one of the hydroxide ligands as the H_2_O solvent evaporates). Therefore, the rinsed precursor is expected to contain only Co­(OH)_2_ and a certain amount of NH_4_NO_3_.

The TG/DSC results (Figure 1a) of the mixture of precursors heated to 500 °C revealed one endothermic (**A**) and one exothermic reaction (**B**), followed by an overall mass decrease. The endothermic peak **A,** with a maximum at 130 °C, can be attributed to the dehydroxylation of cobalt hydroxide^31^ according to eq 3:

3 $$\mathrm{6Co}{\left(\mathrm{OH}\right)}_{2}{+O}_{2}\to {\mathrm{2Co}}_{3}O_{4}{+6H}_{2}O$$

The nitro groups present within the precursor mixture can be regarded as an intrinsic source of oxygen.^32^ The prominent exothermic peak **B,** with a maximum at 244 °C, followed by a significant mass decrease (20%), can be attributed to NH_4_NO_3_ decomposition above 160 °C.^33,34^ The total mass loss is 39.2%, slightly greater than the literature data (34%) for the previously recorded loss of the washed precursor^30^ during the SDM synthesis of Co_3_O_4_. This can be attributed to the leftover NH_4_NO_3_ within the precursor. The TG/DSC results (Figure 1b) of the composite precursor mixture heated to 500 °C revealed a broad endothermic reaction (**A′**) with a maximum at 65 °C, which can be attributed to sample dehydration. As previously discussed, the minor mass stabilization (**B′**) could correspond to Co_3_O_4_ formation and remaining NH_4_NO_3_ decomposition. No other distinguishable energy changes were observed during the overall mass decrease. The total mass loss was 4%, and no stabilization was observed. The TG/DSC results (Figure 1c) for the MS sample revealed a broad endothermic peak (**A″**) with a maximum at 80 °C, which can be attributed to sample dehydration. The sample remained thermally stable overall, with a total mass loss of approximately 0.2%. Considering the mass loss of 39.2% for the cobalt oxide precursor and 0.2% for the MS sample, the Co-MS composite (containing 0.1 g of cobalt oxide per 1 g of MS) would be expected to lose approximately 3.8% of its mass upon heating. This calculated value is in good agreement with the experimentally observed mass loss of around 4%, confirming that the thermal behavior of the Co-MS composite is consistent with its composition.

The crystalline phase and structure of the bulk Co_3_O_4_ sample and the composite were determined using XRD, as shown in Figure 2. The XRD pattern of Co_3_O_4_ reveals a crystalline structure with reflections at 19.0°, 31.3°, 36.9°, 38.6°, 44.8°, 59.4°, and 65.2°, corresponding to the (111), (220), (311), (222), (400), (422), (511), and (440) planes, respectively. These results confirm the presence of crystalline face-centered cubic spinel Co_3_O_4_ (JCPDS No. 01–43–1003).^35–37^ No other peaks were identified. Conversely, the XRD pattern of the Co-MS composite reveals mainly an amorphous structure with only a few low-intensity broad reflections, which cannot be assigned to any of the cobalt oxide polymorphs, indicating their small size and/or low quantity. MS is known to show a hump at 2θ values of 13–32° due to its amorphous nature.^38,39^ The composite sample is thus mainly amorphous, with a diffuse dome at 13–32° 2θ. Two polymorphs of silicon dioxide, cristobalite (JCPDS 00–039–1425), and quartz (JCPDS 01–085–0797), are present in the Co-MS composite sample.

**Figure 2:**
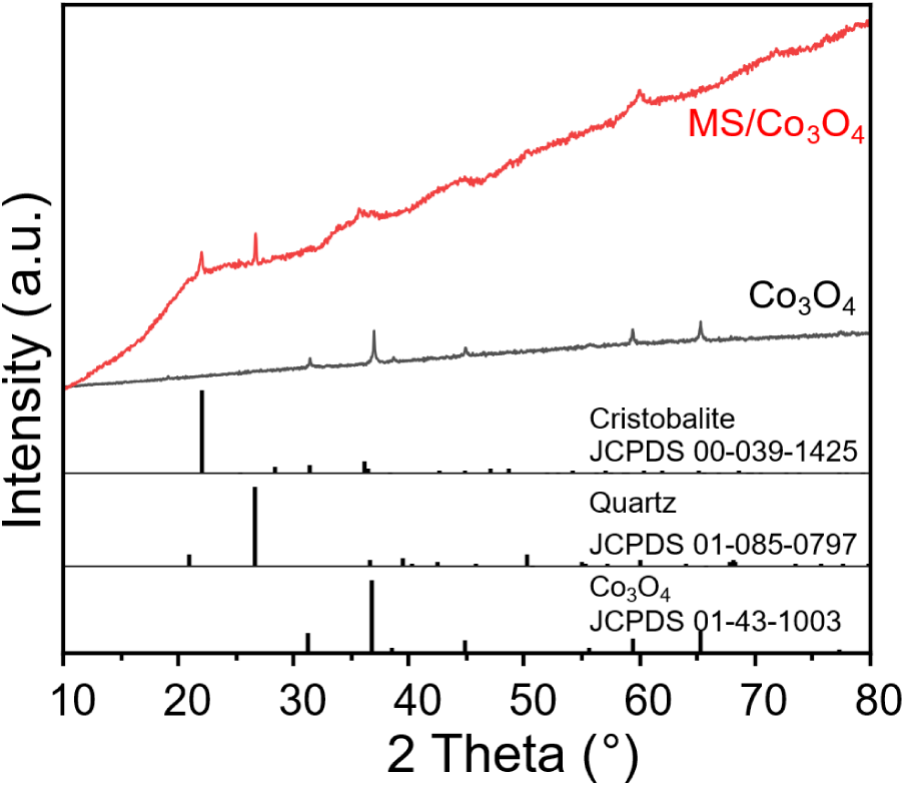
PXRD patterns of the Co_3_O_4_ (black line) and Co-MS composite (red line) after heating at 300 °C for 2 h at a 10 °C·min^–1^ heating/cooling rate. A fully formed single-phase Co_3_O_4_ is present in the oxide sample with a recognized cubic phase. Reflections corresponding to cristobalite and quartz phases are visible in the Co-MS composite, together with a hump at 2θ values of 13–32° due to the MS amorphous nature.

The crystallite size and lattice strain for Co_3_O_4_ obtained from the Williamson–Hall (WH) equation are estimated to be 87 nm and 0.0004, respectively. On the other hand, analysis using Scherrer’s formula showed an average crystallite size of ∼67 nm. The crystallite size determined using the WH plot method is larger than that obtained using the Scherrer equation due to the lattice strain induced by crystallographic imperfections.^40^ The crystallite size of cobalt oxide in the composite could not be determined because of the composite’s amorphous nature and poorly defined reflections.

The micromorphology of the prepared materials was analyzed using SEM, as shown in Figure 3. The distinguishable brighter cobalt oxide nanoparticles—due to larger *z*-number of Co than Si—exhibited a spherical-like morphology (Figure 3b), as previously described.^41^ The Co-oxide nanoparticles only partially cover the MS microspheres and show a very small diameter.

**Figure 3:**
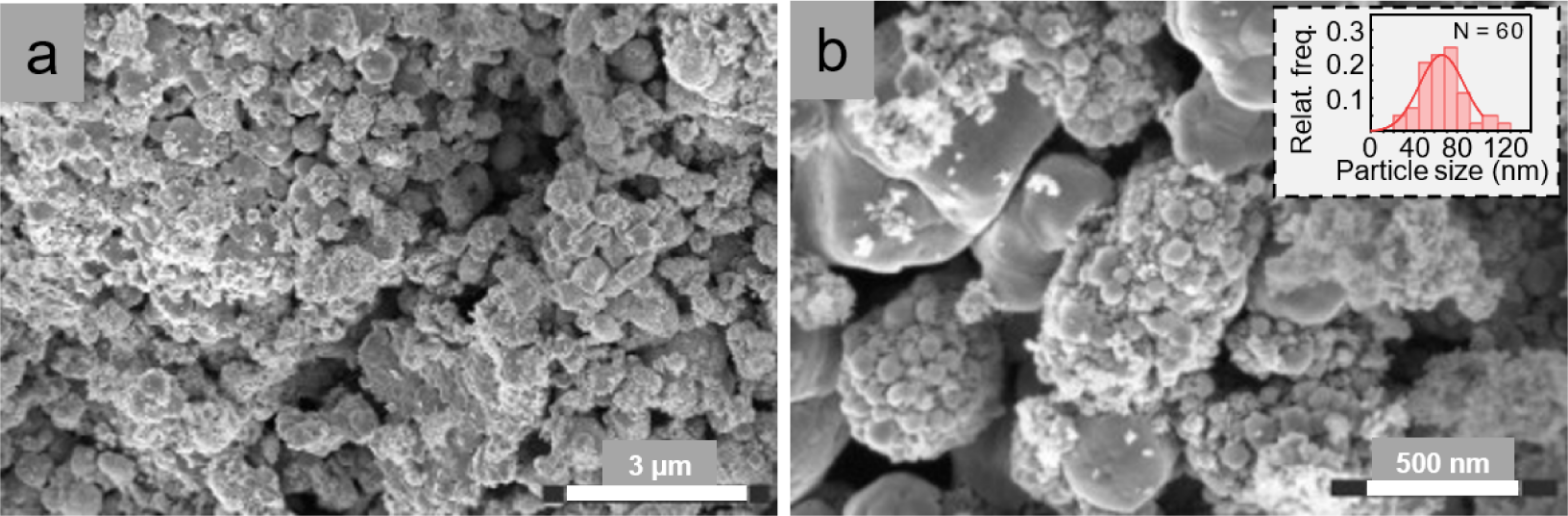
SEM images of the Co-MS composite synthesized by the SDM method, illustrating (a) interparticle voids along with a silica mesostructure and (b) micron-sized MS particles partially covered by nanosized cobalt oxide species. In panel b, the particle size distribution is shown.

Both cobalt oxide species and MS particles tend to aggregate, forming irregularly shaped aggregates. This tendency can be attributed to interparticle forces, including van der Waals interactions.^42^

Further insights into the nature of the composite are shown in TEM and HAADF-STEM images (Figure 4a,b). The spherical structures, with diameters up to 100 nm, correspond to MS covered by cobalt oxide nanoparticles. The brighter regions exhibiting well-defined morphology (Figure 4c) are richer in cobalt, whereas Figure 4d highlights the presence of amorphous carbon on the surface of SiO_2_. The presence of amorphous carbon in MS had been previously confirmed.^14^ SAED patterns of the Co-MS composite, with and without Co-containing nanoparticles, are presented in Figure 4e,f, respectively. The nanoparticles are crystalline, and the corresponding diffraction spots can be indexed to *Fm*3̅*m* cubic CoO, indicating the presence of a complex cobalt oxide structure.

**Figure 4:**
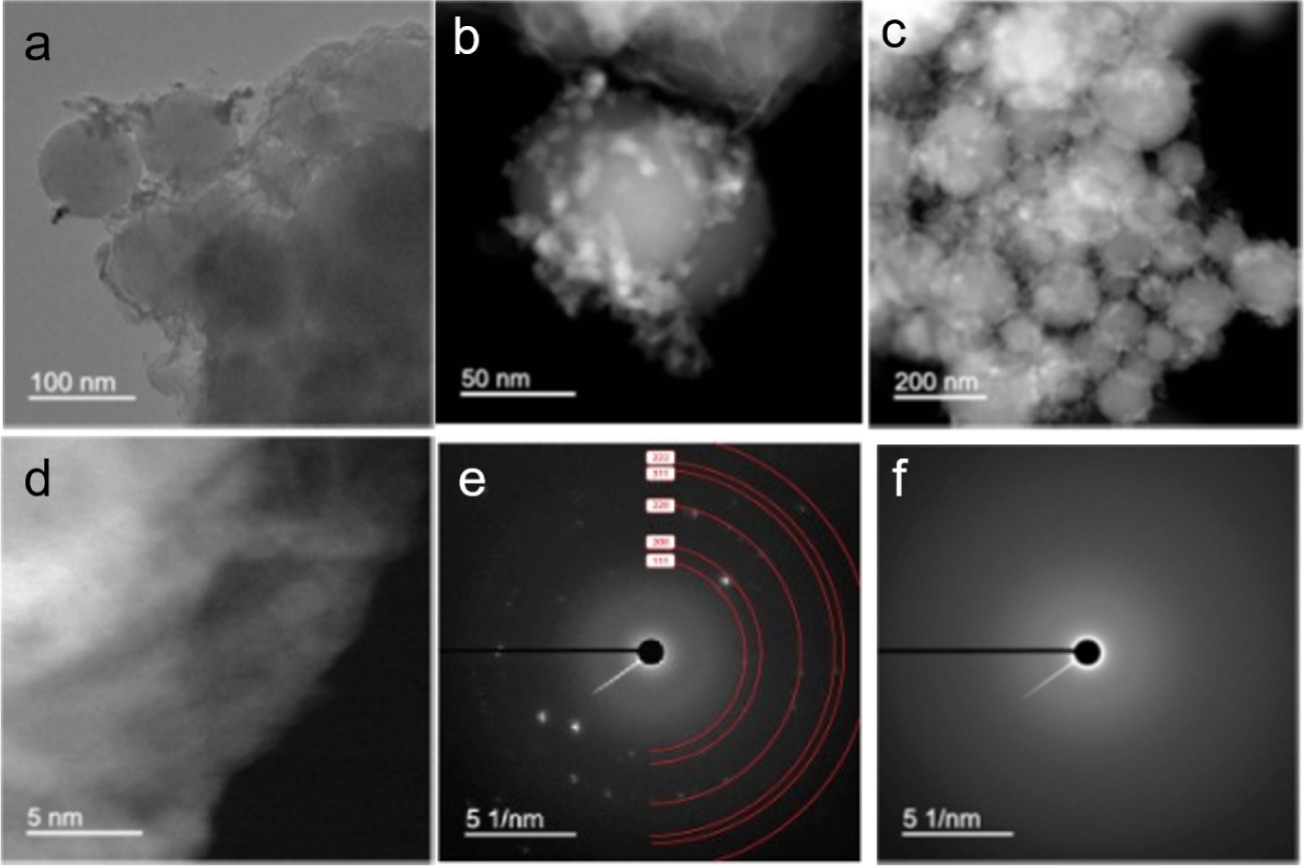
TEM (a) and HAADF-STEM (b) images of the Co-MS composite. Panel c shows dark-field TEM micrographs, with brighter regions with well-defined morphology showing Co-rich domains, while panel d shows a detail of an amorphous region surrounding the SiO_2_ surface. SAED patterns of the composite with and without Co-containing nanoparticles are shown in panels e and f, respectively.

EDXS elemental mapping of the TEM images (Figure 5) shows a uniform distribution of oxygen (Figure 5b) and silicon (Figure 5d). A cluster of cobalt is seen in Figure 5c, which confirms the presence of cobalt nanoparticles. Since the presence of oxygen is rare in this region, the nanoparticles can be ascribed to a combination of cobalt oxide as well as pure cobalt.

**Figure 5:**
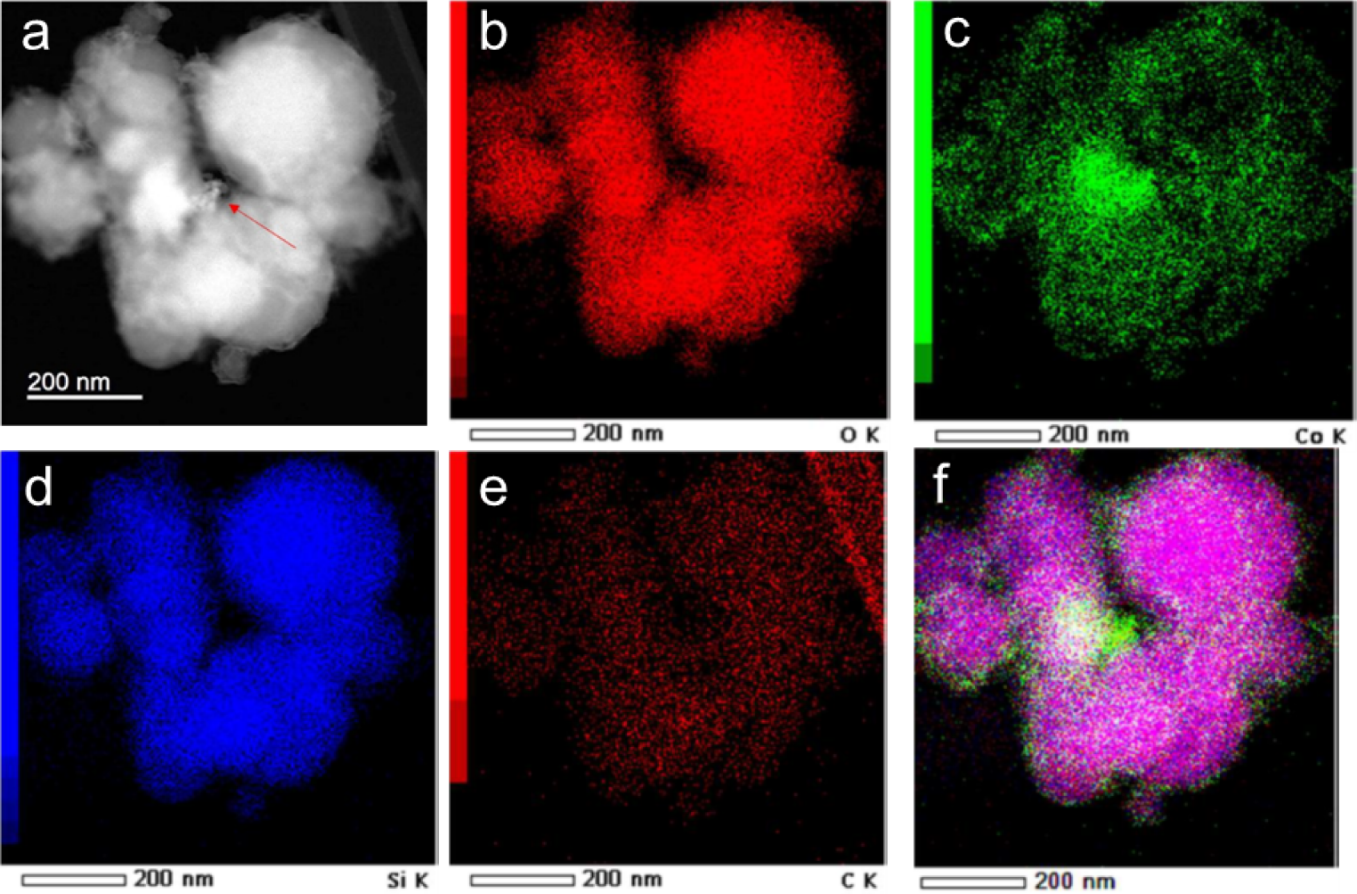
EDXS elemental mapping of the selected region (a) reveals a uniform distribution of O (b) and Si (d). The red arrow in panel a indicates Co nanoparticles, clearly visualized in panel c and shown to be dispersed within the carbon layer (e) that forms a shell around the silica spheres. An overlay of the elemental signals is presented in panel f.

The EELS data (Figure S1, Supporting Information) indicate the presence of the characteristic Co L_2,3_ and O K edges of a spinel cobalt oxide, consistent with Co_3_O_4_. The Co L_3_ edge, when focused on Co-rich nanoparticles, shows a multiple-component structure and the L_3_/L_2_ intensity balance (2.235) that is characteristic of mixed Co^2+^ /Co^3+^ valence rather than a single-valence oxide. This is distinctively different from L_3_/L_2_ ratio for Co of 4.018 found in the area focused on the C-rich shell layer, which is closer to Co metal (L_3_/L_2_ = 3.700). Small energy positions and relative sharpness of the L edges are compatible with a well-crystallized spinel environment, while any minor shifts or broadening likely reflect local variations in stoichiometry, coordination, or specimen thickness. The O K-edge displays a pre-edge feature near the onset (∼529–531 eV) that signals strong O 2p–Co 3d hybridization; higher-energy features correspond to transitions involving Co 4*s*/4p–O states, indicating appreciable covalency of the Co–O bonds. Differences in fine structure between the two spectra can be attributed to variations in local bonding/coordination or to experimental factors (orientation, thickness, or beam-induced reduction), rather than a change in bulk phase. Overall, the spectral fingerprints are most consistent with cobalt oxide with mixed-valence cobalt and significant metal–oxygen covalency.

The FTIR spectra of MS, as well as Co_3_O_4_, and the Co-MS composite synthesized using the SDM are shown in Figure 6. The spectrum of Co_3_O_4_ shows strong peaks at 654 cm^–1^,^30^ and 544 cm^–1^, representing Co–O bond vibrations.^43,44^ The appearance of these bands confirms the XRD data recognizing Co_3_O_4_ spinel nature of the sample.

**Figure 6:**
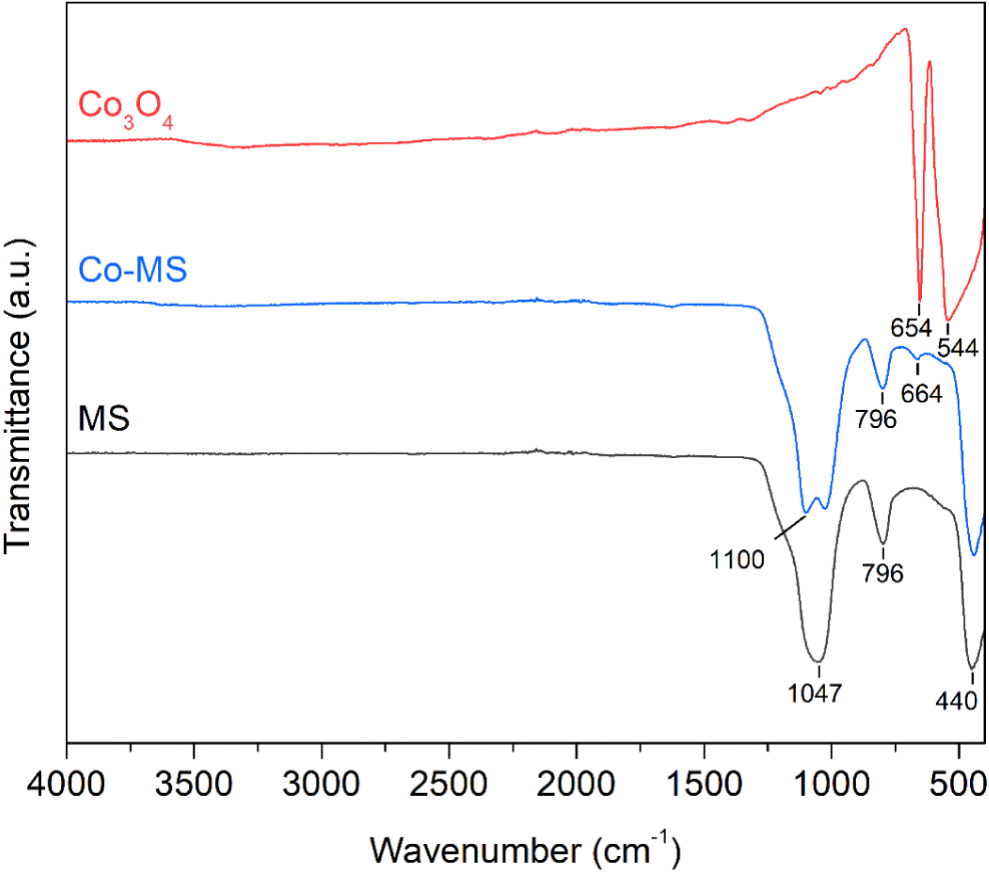
FTIR spectra of Co_3_O_4_ (red), Co-MS composite (blue), and MS (black). The spectra are translated vertically for clarity.

In the composite, the presence of cobalt­(II,III) oxide can only be seen with a weak band at 664 cm^–1^ whose blue shift can be attributed to its incorporation into the silica framework, resulting in confinement, which alters electronic structures and thus affects vibrational modes.^45^ The weaker appearance of this band in the composite is likely due to the limited quantity and quality of Co_3_O_4_ nanoparticles present in the sample. The spectrum of the composite sample is also marked by vibrations of microsilica, with the envelope in the 1000–1300 cm^–1^ region, and bands at 799 and 440 cm^–1^. The latter band is assigned to transverse optical rocking motions (TO_1_ mode). The band near 800 cm^–1^ is due to Si–O–Si symmetric stretching (TO_2_ mode). The highest frequency mode, around 1100 cm^–1^, with the greatest intensity, is assigned to antisymmetric stretching (TO_3_ mode).^46^ This peak is additionally split into two bands in the composite but not in the pristine MS. A band shift toward lower wavenumbers can be due to the presence of higher mass atoms (Co) interacting with SiO_2_ and forming Si–O–Me bonds, as has been demonstrated in several studies lately.^47,48^

The Raman spectra of Co_3_O_4_ and the Co-MS composite are shown in Figure 7. A bulk Co_3_O_4_ sample shows narrow bands at 195, 479, 519, 618, and 689 cm^–1^ corresponding to the Raman-active phonon modes E_g_ + 3F_2g_ + A_1g_, which align well with those reported by other authors for the spinel structure of Co_3_O_4_.^49–51^ The Raman mode at 689 cm^–1^ (A_1g_) is attributed to characteristics of the octahedral sites, and the E_g_ and F_2g_ modes are likely related to the combined vibrations of the tetrahedral site and octahedral oxygen motions. The Co-MS composite shows much broader peaks where SiO_2_ modes superimpose the active modes of Co_3_O_4_. These findings coincide with XRD data, indicating that the 9.1 wt % Co_3_O_4_ phase is shielded in the presence of the silica phase. The wide and low-intensity band at 1598 cm^–1^ is indicative of carbon^52^ and can be attributed to G bands representing the disorder and defects of the local E_2g_ mode of sp^2^ carbon.

**Figure 7:**
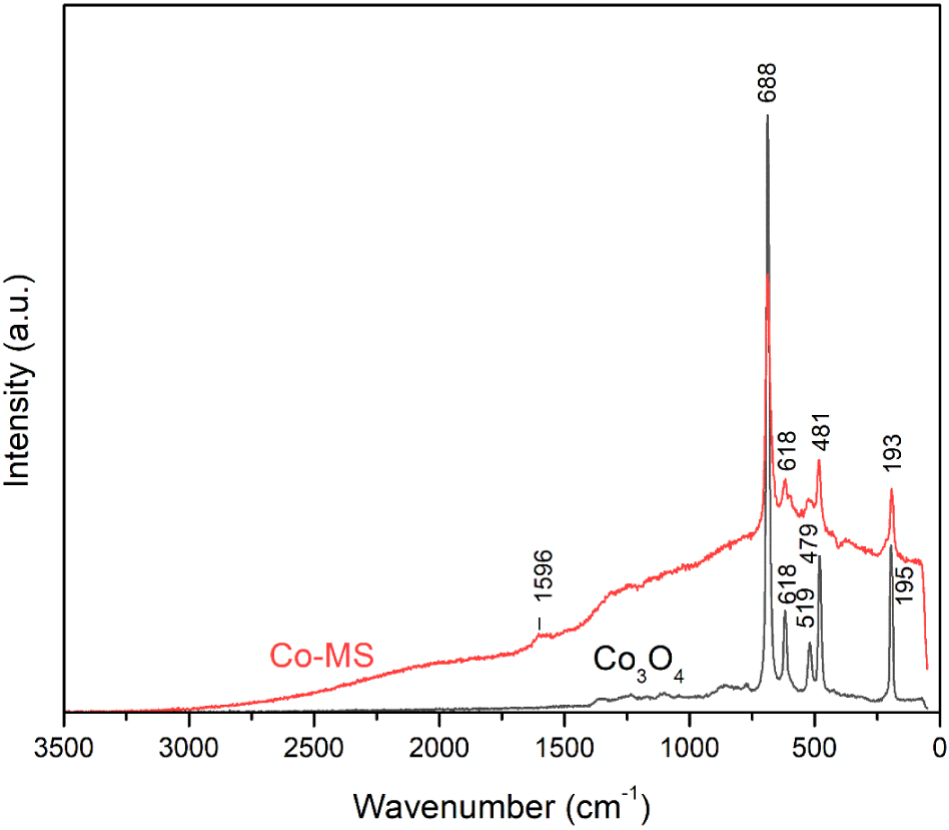
Raman spectra of the Co_3_O_4_ and Co-MS composite synthesized using the SDM at a 785 nm laser intensity of 1 mW·cm^–2^.

Raman results, together with XRD and TEM analyses, show that the Co-MS composite contains Co_3_O_4_ nanoparticles that are sparsely distributed throughout the sample. The composite also exhibits a mixed oxide nature, with the presence of CoO as well as metallic cobalt. Such samples also contain amorphous silica as the support, which includes a small amount of carbon.

The amorphous carbon in microsilica (silica fume) originates as an impurity from the carbothermic reduction process used in its production. During the high-temperature reduction of quartz (SiO_2_) with carbonaceous reductants (coke, coal) in electric arc furnaces, unreacted elemental carbon is entrained with the silica vapor that condenses into fume particles. This free carbon typically constitutes a few percent of the silica fume’s mass and is a process-derived impurity rather than a structural component of the silica network.^53,54^ The carbon content varies with manufacturing parameters, including furnace temperature, reductant type, and product being produced. Since the amorphous carbon content is relatively low (typically 1–5 wt %) and may overlap with other mass loss events (adsorbed water, surface hydroxyl condensation), potentially making it difficult to resolve as a distinct feature in the thermogram (Figure 1).

To get insight into the pore structure of such samples, the nitrogen adsorption–desorption isotherms of the Co_3_O_4_, Co-MS composite, and MS sample were measured (Figure 8). The isotherms of the Co_3_O_4_ sample and the composite are type III isotherms, as defined by the IUPAC, while the MS sample displays a Type II isotherm, characteristic of macroporous adsorbents.^55^ Type III isotherm indicates nonporous or macroporous solids, e.g., many oxides, etc.^30,56^ Hystereses in the isotherms of Co_3_O_4_ and the composite sample correspond to the H3 type.

**Figure 8:**
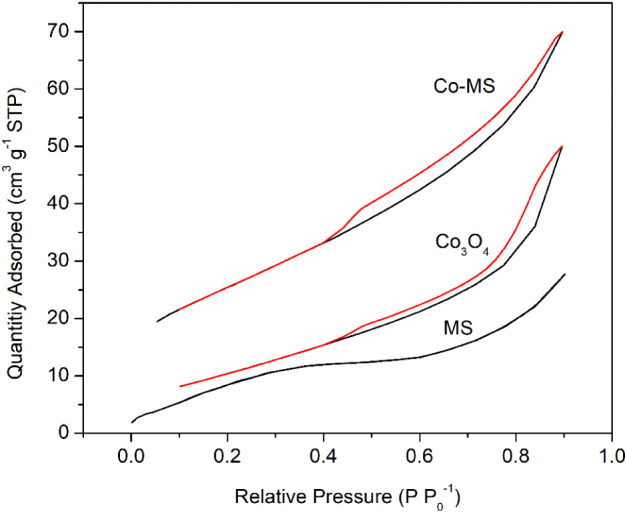
Nitrogen adsorption (black lines) and desorption (red lines) isotherms for MS, Co_3_O_4_, and Co-MS (S3) composite synthesized using the SDM.

Owing to the relatively low BET surface area (Table 1), the interparticle voids within agglomerates create a porous structure, which primarily governs the material’s nitrogen adsorption behavior.

**Table 1:** Results of Nitrogen Adsorption of the Co_3_O_4_, MS, and Co-MS Composite^a^

Sample	Specific surface area (m^2^·g^–1^)	Pore volume (cm^3^·g^–1^)	Pore size (nm)
Co_3_O_4_	43	0.077	7.3
MS	24	0.08	4.5
Co-MS composite	91	0.108	4.8

a The Co-MS composite shows a marked enhancement in its textural properties.

The specific surface area of the synthesized Co_3_O_4_ agrees well with values reported for chemically precipitated Co_3_O_4_ prepared at similar temperatures,^57^ while some studies^24^ reported lower *S*_BET_ and similar average pore sizes, considering the mixture of cobalt oxides. While MS itself shows limited surface area development (with restricted pore volume and narrow pore size distribution), the composite exhibits considerably enhanced textural properties. This improvement probably arises from the uniform dispersion of cobalt oxide nanoparticles throughout the MS scaffold, which simultaneously lowers the nanoparticle aggregation and creates an interconnected porous network. Thus, the observed variations in composite surface area originate from inherent MS characteristics and specific synthesis parameters.^56^

Using the Tauc method (Figure 9) and considering a direct band structure,^58^ the bandgap energy value (Eg) in the Co_3_O_4_ sample was determined to be 2.15 eV. This aligns well with the literature values for Co_3_O_4_.^59,60^ The optical band gap of 2.15 eV can be associated with the O^2–^→Co^2+^ charge transfer process (excitation from the valence band (VB) to the conduction band (CB) of ions in octahedral coordination spheres). In fact, there is a second absorption observable at 1.45 eV, corresponding to d–d of Co^3+^ and Co^2+^ ions in the tetrahedral coordination spheres.^19^

**Figure 9:**
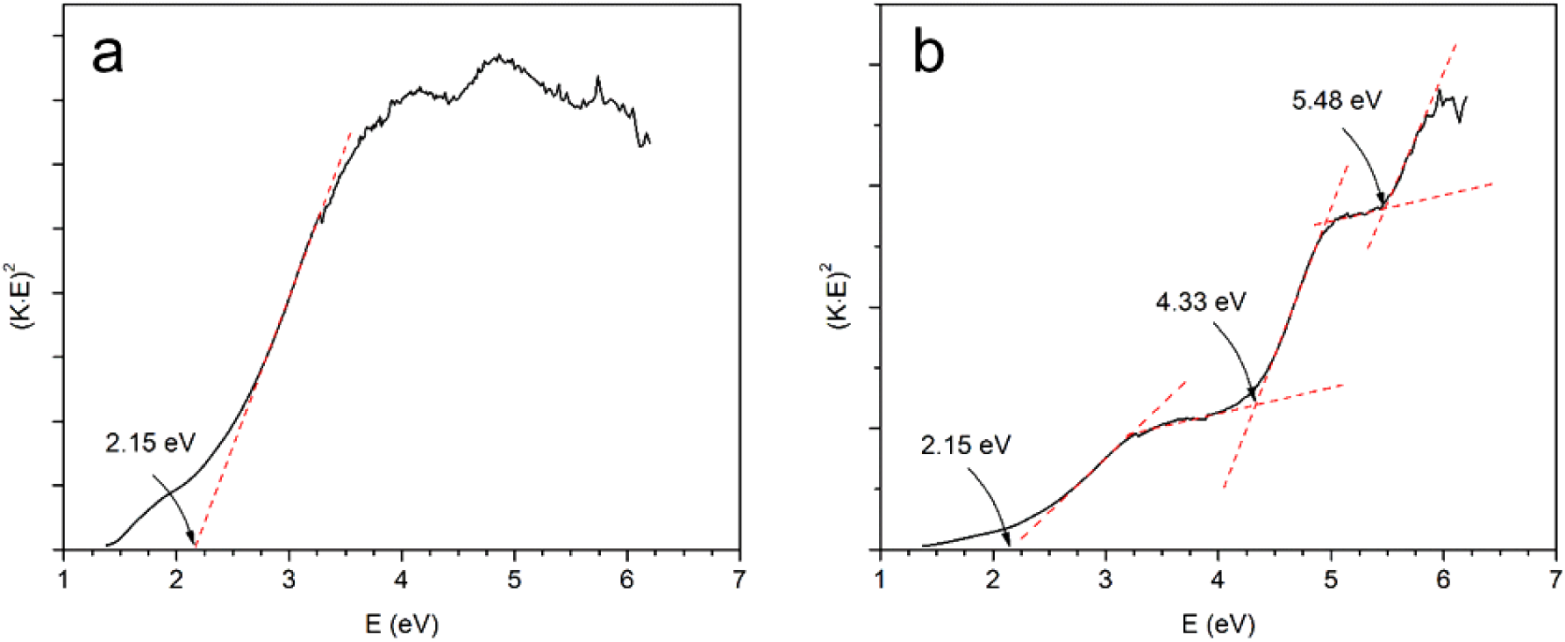
Tauc plot diagrams for direct bandgap energy values of a) Co_3_O_4_ and multiple direct Eg values for the b) Co-MS composite synthesized using the SDM.

The Co-MS composite showed multiple indirect bandgap energies of 2.15, 4.33, and 5.48 eV, from the optical contributions of multiple components in the sample. The latter belongs to the Eg of MS.^61^ The data indicate that the Co_3_O_4_ phase remained unchanged when incorporated with MS and is responsible for the visible part absorption of the composite sample. Photocatalysts with 2.0–2.5 eV band gaps effectively absorb visible light (400–700 nm), aligning with a large part of the sunlight spectrum.^62^

In a previous research on the photocatalytic performance of MS-based Co_3_O_4_/MCM-41 green nanocomposites,^56^ the bandgap energy values were determined to be 1.97 eV (Co_3_O_4_) and 1.53 eV (Co_3_O_4_/SF-MCM-4). This shift was related to the substrate’s ability to provide cobalt oxide with highly light-exposed properties by consistently orienting Co_3_O_4_ within its mesoporous structure. A similar influence is also expected with the Co-MS composite. In another study,^63^ the bandgap energies of MS and SiO_2_–Co_3_O_4_ composites were 2.45 and 3.0 eV, respectively.

In our work, the direct bandgap energy of 2.15 eV in the Co_3_O_4_ sample is within the reported range. The multiple direct band gaps observed in the Co-MS composite can thus be attributed to the presence of distinct domains of Co_3_O_4_ and the silica substrate. This effect was also noted previously in the case of the MoS_2_–MoO_3‑x_ heterojunction.^64^ The existence of multiple direct bandgaps may contribute to the photocatalytic activity of the material, as described previously.^65^

The DRS spectra, transformed according to the Kubelka–Munk theory, revealed three absorption bands for Co_3_O_4_ sample located at 383, 531, and 677 nm (Figure 10a). The band at around 531 nm is commonly ascribed to the ligand field ^4^A_2_(F) → ^4^T_1_(P) transition of Co^2+^ ions in tetrahedral coordination.^66–68^ Thus, the spectra indicate the formation of Co_3_O_4_ particles.

**Figure 10:**
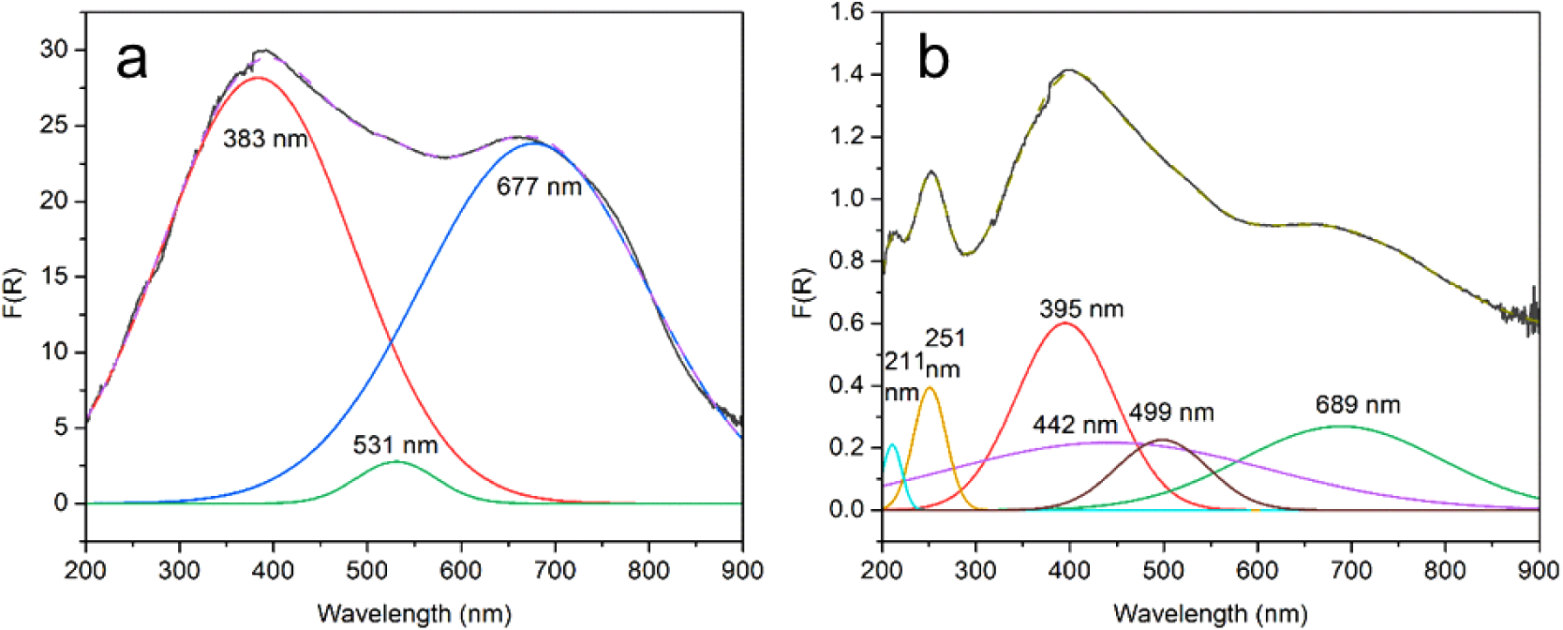
Deconvolution and fitting of the DRS spectra, transformed according to the Kubelka–Munk theory for a) Co_3_O_4_ and for b) the Co-MS composite synthesized using the SDM. The measured spectra are shown in black by full lines, while the final fitted spectra are displayed by dashed lines. The corresponding absorption bands are presented separately, with the peak positions indicated.

Accordingly, spectrum fitting for the Co-MS composite (Figure 10b) revealed the existence of six peaks at 211, 251, 395, 442, 499, and 689 nm. In fact, the two wide VIS envelopes of bands, 400 and 700 nm, are related to the presence of cobalt mixed valence oxide [Co^III^_2_Co^II^O_4_][Bibr ref68] and to Co^3+^ ions in an octahedral environment from well-ordered Co_3_O_4_ species.^66^ According to Yeshchenko et al.,^69^ the peak at around 685 nm can be attributed to the light absorption by Co^2+^ ions in the silica matrix in tetrahedral coordination, while the peak at around 490 nm corresponds to light absorption by Co^2+^ ions in the silica matrix in octahedral coordination. The same authors implied that the band with a maximum at 230 nm is caused by light absorption by CoO nanoparticles dispersed in the silica matrix, while the band at 380 nm can be attributed to the absorption of light by Co_3_O_4_ nanoparticles.

### Photocatalytic Properties

The photocatalytic experiment conducted under sunlight using bulk Co_3_O_4_ showed that cobalt oxide alone could only remove 20.2% of MB in 3 h and remained at this level regardless of the Co_3_O_4_ photocatalyst mass used (50, 100, or 150 mg). For example, when using the largest amount of Co_3_O_4_ (150 mg), the degradation rate reached 20.2%, which was close to the removal by MS adsorption after 3 h (Figure 11) in dark conditions. On the other hand, photolysis of MB showed slightly higher values, reaching ∼35% removal after 3 h of illumination. No synergy was observed with the MS sample, meaning that adding illumination to this sample (45.9% removal after 3 h) did not exceed the summation of the dark experiment and photolysis (theoretically, 60% removal after 3 h). This implies that MS functioned as an efficient support for the adsorption of MB, allowing for the degradation of some of the adsorbed MB molecules by sunlight. Illumination may also have caused some of the adsorbed MB molecules to be rearranged on the surface and/or degraded, thus allowing better packing of MB onto the surface and resulting in higher and faster adsorption than in the dark experiment.

**Figure 11:**
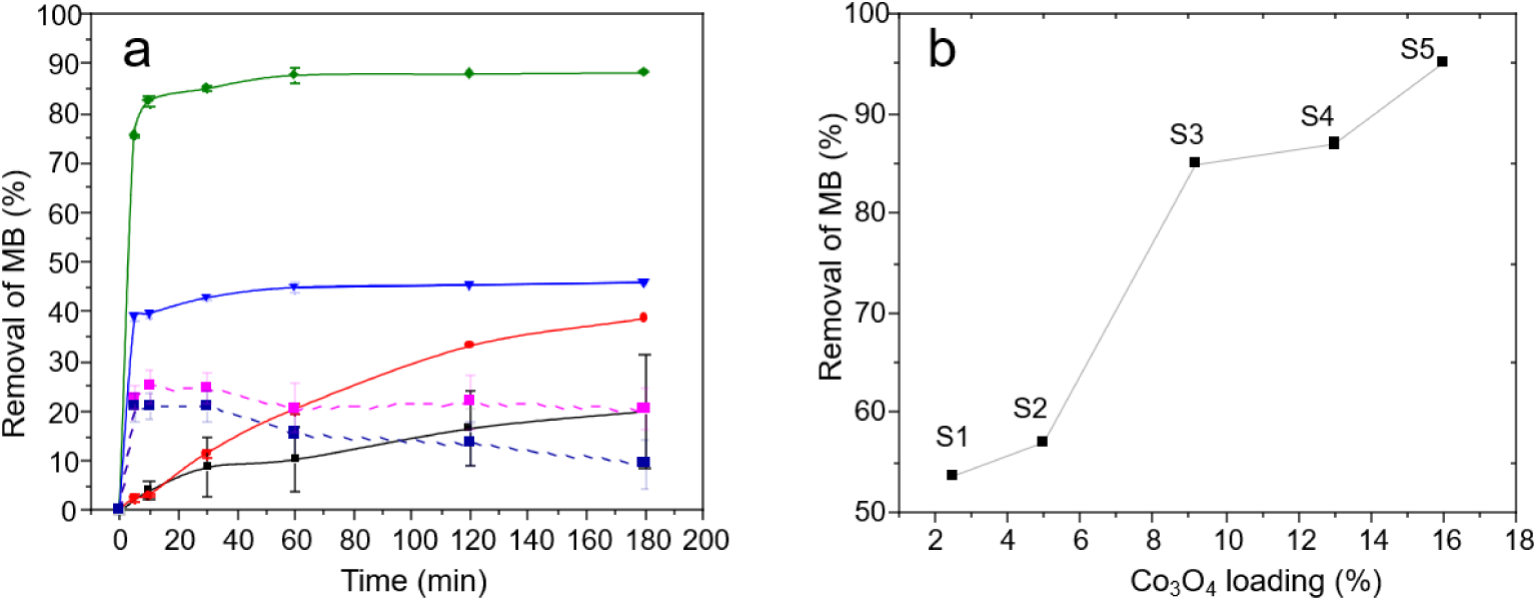
MB removal over 180 min under different conditions (a). Photocatalytic tests (solid lines): ⧫—Co-MS composite, ▼—MS, ■ – Co_3_O_4_, and ●—photolysis. Adsorption tests without illumination (dashed lines): pink ■—MS and dark blue ■—S3 (Co-MS composite). Each experiment used 100 mL of 10 mg L^–1^ MB with either 100 mg of S3 or MS, 150 mg of Co_3_O_4_, or no catalyst. Dependence of MB removal on the Co_3_O_4_ mass loading (b) in the Co-MS composites (S1, S2, S3, S4, and S5 samples) after 60 min of sunlight illumination. Each experiment used 100 mL of 10 mg·L^–1^ MB with 100 mg of catalyst.

On the other hand, for the Co-MS composites, an increase in the Co_3_O_4_ weight fraction in the composite increased the degree of MB removal (Figure 11b) within 1 h. The degradation achieved with the S5 composite was impressive, reaching 95.4%. The S3 composite, with a Co_3_O_4_ loading of 9.1 wt % (0.1 g Co_3_O_4_ per 1 g MS), was identified as the optimal formulation for the final photocatalytic experiments, due to its relatively low Co_3_O_4_ fraction while achieving a high degree of MS removal. Although a higher mass loading of Co_3_O_4_ in the mixture resulted in a higher degree of degradation, a lower mass fraction of Co_3_O_4_ was deemed optimal because of economic and environmental considerations and was thus subjected to the characterizations described above.

After 60 min, the degree of MB removal with the S3 composite was 87.5%, approximately four times greater than that of MB photolysis (20.4%) and almost nine times greater than pure Co_3_O_4_ (10%), indicating synergism between MS and Co_3_O_4_. Since there was a fast decrease of MB concentration in the first hour, similar to that of MS adsorption (Figure 8a), this implies that the synergism might stem from the effect of MS in attracting the dye molecules to the Co_3_O_4_ active sites, where they can be efficiently degraded and later removed as the reaction proceeds. This notion is corroborated with observation that pure Co_3_O_4_ showed slow MB removal, reaching only 10% in 1 h, indicating difficulties in the mass transport of MB molecules to catalyst’s active sites—problem which was effectively circumvented with the addition of MS. Data from SEM and N_2_-sorption additionally confirm this thesis since higher surface area was obtained in the S3 sample, suggesting a deaggregation effect of MS in the composite.

Furthermore, the photocatalytic degradation of methylene blue followed apparent first-order kinetics under all investigated conditions. The apparent pseudo-first-order rate constants and correlation coefficients over 180 min were 0.0028 min^–1^ for MB alone (R^2^ = 0.9739), 0.0017 min^–1^ (R^2^ = 0.2933) in the presence of silica fume, and 0.0015 min^–1^ (R^2^ = 0.8364) for pure Co_3_O_4_, indicating that neither silica fume nor Co_3_O_4_ individually enhanced MB photodegradation compared to photolysis. In contrast, the Co-MS composite exhibited a significantly higher rate constant (0.0074 min^–1^, R^2^ = 0.4149), demonstrating a clear synergistic effect. However, the reaction exhibits fast initial degradation immediately after *t* = 0 (>80% within the first 15 min), followed by a slower stage, indicating deviation from simple first-order kinetics. This is also reflected in the relatively low correlation coefficients for Co-MS, MS, and Co_3_O_4_, showing that the linear first-order model does not fully describe the reaction behavior.

In a study by Zha et al.^22^ on the facile synthesis of Co_3_O_4_ nanoparticle-functionalized mesoporous SiO_2_ for the catalytic degradation of MB in aqueous solutions, the pseudo-first-order rate constants of the Co-SiO_2_ photocatalysts were determined to be 0.055, 0.059, 0.058, and 0.049 min^–1^ for Co/SiO_2_ ratios of 0.17, 0.08, 0.04, and 0.02, respectively. The kinetic curves of MB degradation over Co-SiO_2_ catalysts were monitored over a 60 h time frame. In a related study^62^ investigating the photocatalytic degradation of MB under natural sunlight using iron titanate nanoparticles synthesized via a modified sol–gel method, the pseudo-first-order rate constants were reported as 4.65 × 10^–4^, 0.00356, 0.00612, and 0.016 min^–1^ at initial pH values of 3, 7.19, 9, and 11, respectively. The corresponding correlation coefficients (R^2^) for the linear fits were 0.753, 0.977, 0.979, and 0.994, indicating improved kinetic fitting at higher pH values. The kinetic curves of MB degradation over iron titanate nanoparticle catalysts were monitored over a 240 min time frame. In another study focusing on the synergistic degradation of methylene blue using a novel Fe–Co bimetallic catalyst supported on waste silica,^70^ a pseudo-first-order rate constant of 0.065 min^–1^ was achieved over 60 min of operation.

When compared to the literature, our Co-MS system shows moderate activity, higher than bare MS (0.0007 min^–1^) and Co_3_O_4_ (0.0016 min^–1^), but lower than previously reported Co_3_O_4_–SiO_2_ catalysts (0.049–0.059 min^–1^) and Fe–Co bimetallic catalysts on waste silica (0.065 min^–1^). Iron titanate nanoparticles (0.00465–0.016 min^–1^ depending on pH) show activity comparable to or slightly higher than our Co-MS at alkaline conditions, but lower at acidic or neutral pH. These comparisons highlight the strong influence of metal dispersion, support structure, reaction environment, and very fast initial kinetics on the observed degradation rates.

Additionally, the superior photocatalytic performance of Co-MS could be attributed to synergistic effects among the mixed-valence cobalt species (Co^0^, Co^2+^, Co^3+^). Metallic cobalt facilitates strong molecular adsorption and e^–^ transfer due to its d-band center position near the Fermi level, while Co^2+^ acts as a Lewis acid site, promoting substrate polarization and stabilizing radical intermediates through single-electron transfer mechanisms. Highly oxidized Co^3+^ exhibits strong electrophilicity, activating lattice oxygen to generate reactive oxygen species (O^–^ or O_2_^–^) that drive photocatalytic oxidation.^71^ This multivalent synergy enables flexible electron transfer pathways and rapid Co^3+^ /Co^2+^ redox cycling, which are critical for efficient charge separation and ROS generation under solar irradiation. The coexistence of these oxidation states, as confirmed by XPS analysis, creates complementary active sites that collectively enhance the photocatalytic degradation of MB beyond what would be achieved by single-phase Co_3_O_4_ alone.

## Conclusions

Cobalt-functionalized waste silica (Co-MS) was prepared using a solvent-deficient method (SDM). Comprehensive characterization confirmed the formation of multiple-valence-state cobalt nanoparticles, i.e., Co, CoO, and Co_3_O_4_, on the waste silica support. The Co-MS composite exhibited remarkable photocatalytic efficiency under solar irradiation, degrading 87.5% of MB within 1 h, synergistically exceeding the performance of bulk Co_3_O_4_ (20.2% after 3 h) and MB photolysis (20.4% MB removal).

The enhancement was attributed to several factors: (i) composite’s larger surface area (91 m^2^ g^–1^ vs 43 m^2^ g^–1^) and larger pore volume, (ii) the presence of additional cobalt oxide nanoparticles, and (iii) improved light absorption due to multiple bandgap energies (2.15, 4.33, and 5.48 eV).

A major highlight of this work is the successful utilization of Co-MS, where this approach not only enhances photocatalytic performance but also provides an eco-friendly and cost-effective solution for waste valorization, contributing to circular economy principles. These findings underscore the potential of waste-derived Co-MS composites as efficient solar-driven photocatalysts for environmental remediation. Future studies could focus on optimizing the composite for real wastewater treatment and scaling up production for industrial applications.

## Supplementary Material



## Notes

Electron energy loss spectroscopy (EELS) acquired during TEM analysis for the S3 Co-MS composite (PDF)
