# Supplementary material for: From Waste to Catalyst: Cobalt-Functionalized Silica as a Photocatalyst for Water Purification
Source: ACS Omega. 2026 Jul 17;11(29):43203–14. doi: 10.1021/acsomega.6c00378 (PMC13425333; doi:10.1021/acsomega.6c00378)
Supplement: Supplementary file 1 [file ao6c00378_si_001.pdf]

Supplementary material

for

From Waste to Catalyst: Cobalt-Functionalized Silica as a Photocatalyst for Water Purification

Saša Zeljković[1,1,], Andraž Šuligoj[2,3,*], Milica Kosić[1], Goran Dražić[4], Alenka Ristić[2], Nataša Zabukovec Logar[2,5], Nataša Novak Tušar[2,5]*

[1] University of Banja Luka, Faculty of Natural Sciences and Mathematics, Department of Chemistry, Mladena Stojanovica 2, 78 000 Banja Luka, Bosnia and Herzegovina

[2] Department of Inorganic Chemistry and Technology, National Institute of Chemistry, Hajdrihova 19, SI-1001 Ljubljana, Slovenia

[3] Faculty of Chemistry and Chemical Technology, University of Ljubljana, Večna pot 113, SI-1000 Ljubljana, Slovenia

---

* Corresponding authors. E-mail: andraz.suligoj@ki.si (Andraž Šuligoj), sasa.zeljkovic@pmf.unibl.org (Saša Zeljković)

[4] Department of Materials Chemistry, National Institute of Chemistry, Hajdrihova 19, SI-1001 Ljubljana, Slovenia

[5] Graduate School, University of Nova Gorica, Vipavska 13, SI-5000 Nova Gorica, Slovenia

EELS (Electron energy loss spectroscopy) data from TEM imaging shows L3/L2 cobalt ratio is different for Co present in particle and in carbon layer indicating different valence state. Oxygen may originate from SiO_2 microspheres. In EELS of nanoparticle there is an intense white line before the O K edge.

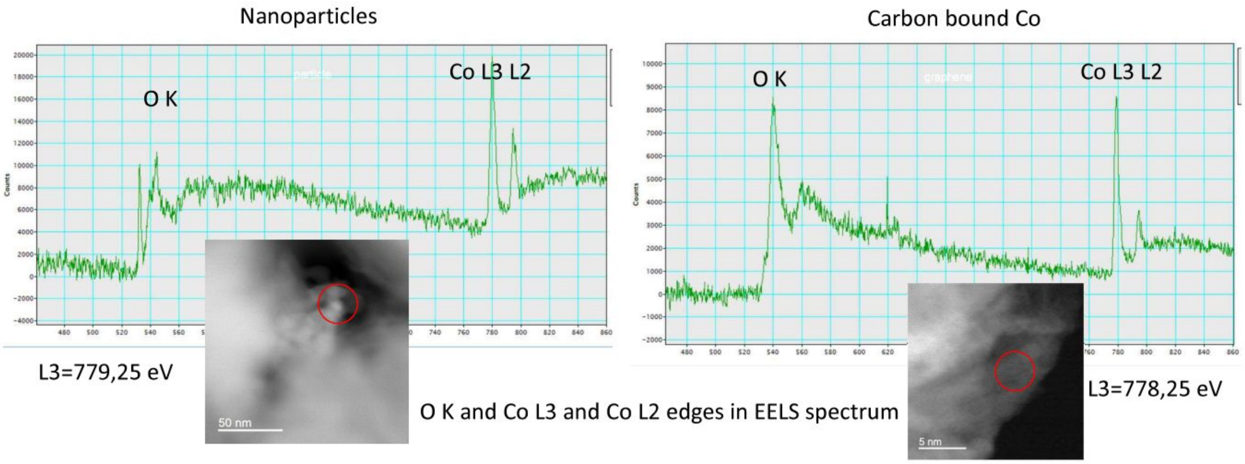

Figure S1. Electron energy loss spectroscopy (EELS) acquired during TEM analysis on the S3 Co-MS composite. The Co L3/L2 intensity ratio differs between cobalt nanoparticles and cobalt present in the carbon layer, indicating different cobalt valence states. The detected oxygen signal

may originate from the SiO_2 microspheres. The EELS spectrum of the nanoparticle also exhibits an intense white line immediately preceding the O K-edge. Insets show the corresponding TEM images from the regions where the EELS spectra were acquired.
